# A low protein diet during pregnancy provokes a lasting shift of hepatic expression of genes related to cell cycle throughout ontogenesis in a porcine model

**DOI:** 10.1186/1471-2164-13-93

**Published:** 2012-03-16

**Authors:** Michael Oster, Eduard Murani, Cornelia C Metges, Siriluck Ponsuksili, Klaus Wimmers

**Affiliations:** 1 Research Unit Molecular Biology, Leibniz Institute for Farm Animal Biology (FBN), Dummerstorf, Germany; 2 Research Unit Physiology of Nutrition, Leibniz Institute for Farm Animal Biology (FBN), Dummerstorf, Germany; 3 Research Group Functional Genomics, Leibniz Institute for Farm Animal Biology (FBN), Dummerstorf, Germany

## Abstract

**Background:**

In rodent models and in humans the impact of gestational diets on the offspring's phenotype was shown experimentally and epidemiologically. Adverse environmental conditions during fetal development provoke an intrauterine adaptive response termed 'fetal programming', which may lead to both persistently biased responsiveness to extrinsic factors and permanent consequences for the organismal phenotype. This leads to the hypothesis that the offspring's transcriptome exhibits short-term and long-term changes, depending on the maternal diet. In order to contribute to a comprehensive inventory of genes and functional networks that are targets of nutritional programming initiated during fetal life, we applied whole-genome microarrays for expression profiling in a longitudinal experimental design covering prenatal, perinatal, juvenile, and adult ontogenetic stages in a porcine model. Pregnant sows were fed either a gestational low protein diet (LP, 6% CP) or an adequate protein diet (AP, 12% CP). All offspring was nursed by foster sows receiving standard diets. After weaning, all offspring was fed standard diets *ad libitum*.

**Results:**

Analyses of the hepatic gene expression of the offspring at prenatal (94 *dies post conceptionem*, dpc) and postnatal stages (1, 28, 188 *dies post natum*, dpn) included comparisons between dietary groups within stages as well as comparisons between ontogenetic stages within diets to separate diet-specific transcriptional changes and maturation processes. We observed differential expression of genes related to lipid metabolism (e.g. Fatty acid metabolism, Biosynthesis of steroids, Synthesis and degradation of ketone bodies, FA elongation in mitochondria, Bile acid synthesis) and cell cycle regulation (e.g. Mitotic roles of PLK, G1/S checkpoint regulation, G2/M DNA damage checkpoint regulation). Notably, at stage 1 dpn no regulation of a distinct pathway was found in LP offspring.

**Conclusions:**

The transcriptomic modulations point to persistent functional demand on the liver towards cell proliferation in the LP group but not in the AP group at identical nutritional conditions during postnatal life due to divergent 'programming' of the genome. Together with the observation that the offspring of both groups did not differ in body weight but in body composition and fat content, the data indicate that the activity of various genes led to diverse partitioning of nutrients among peripheral and visceral organs and tissues.

## Background

Pregnancy and fetal development are periods of rapid growth and cell differentiation when mother and offspring are vulnerable to changes in dietary supply. Adverse environmental conditions during fetal development provoke an intrauterine adaptive response termed 'fetal programming', which may lead to both persistently biased responsiveness to extrinsic factors and permanent consequences for the organismal phenotype [1-6]. Due to developmental plasticity, environmental factors induce altered expression of the genome and ultimately modify the offspring's phenotype [7]. In various human and animal studies a gestational low protein intake during pregnancy was accompanied by low birth weight offspring, which was subsequently predisposed for metabolic disorders and alterations in body composition [6,8-10]. Interestingly, epidemiological studies in women showed that maternal malnutrition during pregnancy can result in fetal growth retardation [11,12].

In order to contribute to a comprehensive inventory of genes and functional networks that are targets of nutritional programming initiated during fetal life, we applied whole-genome microarrays for expression profiling in a longitudinal experimental design covering prenatal, perinatal, juvenile and adult ontogenetic stages in a porcine model. On an isoenergetic basis, pregnant sows were fed either a gestational low protein diet (LP, 6% CP) or an adequate protein diet (AP, 12% CP) to investigate the effects on hepatic gene expression in their fetuses and offspring. The experiment comprises a valuable model especially for 'fetal' programming, because cross-fostering enabled assessment of solely the nutritional effects during gestation. Moreover, due to the similarity in metabolism, physiology, anatomy and genome the study is also a beneficial model for nutritional programming in humans [13,14]. Thus the experimental data will complement previous findings from rodent models and epidemiological human data. In particular, the low protein diet provides a model for prenatal dietary undersupply and exposure to famine that regrettably still burdens a considerable proportion of the human population.

In our experiment the porcine offspring, which was exposed to an undersupply of protein during fetal development but had appropriate postnatal dietary conditions, was able to broadly adapt in terms of their body weight. In fact, newborns from sows that received a low protein supply during gestation had a significantly lower birth weight, a lower body fat content, reduced size and number of adipocytes and muscle fibres than newborns of the control group. At weaning (28 dpn) offspring of the LP group showed slight but significant higher fat content and adipocyte size, and still lower muscle fibre numbers. But neither body weight at weaning nor body weight at 188 dpn differed significantly among offspring of the LP and the AP group, whereas visceral and subcutaneous fat content remained higher in LP than in AP during postnatal life [15-17]. Here the focus is on the hepatic transcriptomic response. We present that the hepatic expression profiles showed considerable modulation during prenatal and postnatal stages, i.e. in acute and delayed response to the nutritional stimulus. Nutritional fetal programming becomes apparent as an altered hepatic expression of genes related to cell cycle and cell maintenance, and lipid, ketone body, and amino acid metabolism, indicating different functional demands and replies of the liver in both experimental groups under identical nutritional conditions *post natum*.

## Results

We performed a longitudinal holistic study of the hepatic transcriptome of offspring of dams fed either an experimental low protein diet (LP) or an adequate protein diet (AP) throughout gestation, in order to obtain a comprehensive picture of genes and functional networks that are sensitive to fetal nutritional programming using a porcine model. We investigated the offspring's hepatic gene expression at 94 dpc, 1 dpn, 28 dpn and 188 dpn by 24 k-microarray analysis. In total we found 12,650 probe-sets expressed at stage 94 dpc (1 dpn: 12,005; 28 dpn: 12,307; 188 dpn: 11,784) according to MAS5 analysis. Further filtering based on the variability of expression of probe-sets revealed 7,937 probe-sets for further analysis at stage 94 dpc (1 dpn: 9,099; 28 dpn: 8,250; 188 dpn: 8,943). These probe-sets represent 5,887 genes at stage 94 dpc (1 dpn: 6,965; 28 dpn: 6,387; 188 dpn: 6,958), according to the recent annotation [18]. In order to identify molecular pathways affected by the gestational diets we first analysed differential expression between the dietary groups within each stage separately. The different dietary exposure of the offspring during prenatal development can be expected to cause slight shifts of the developmental age of the offspring that may be reflected by subtle changes of the transcriptome and could hamper the identification of direct effects of the gestational diets on the hepatic expression. Secondly, we analysed the differences among both experimental groups regarding the more long-term and more pronounced changes of expression patterns between the adjacent stages. In total we found 13,357 probe-sets expressed within 94 dpc and 1 dpn (1 dpn and 28 dpn: 13,259; 28 dpn and 188 dpn: 12,637) according to MAS5 analysis (Table 1). After the filtering steps described above, 10,293 probe-sets were detected within 94 dpc and 1 dpn (1 dpn and 28 dpn: 10,317; 28 dpn and 188 dpn: 8,892). These probe-sets represent 7,697 genes within 94 dpc and 1 dpn (1 dpn and 28 dpn: 7,758; 28 dpn and 188 dpn: 6,879). Notably, q-values between ontogenetic stages within diet were remarkable lower (*q *≤ 0.05) than between diets within stage (*q *≤ 0.25).

**Table 1:** No. of expressed probe-sets, filtered probe-sets, and regulated probe-sets of LP and AP offspring at distinct developmental stages and periods

No. of expressed probe-sets	No. of filtered probe-sets	No. of total regulated probe-sets	No. of regulated probe-sets private to LP offspring	No. of regulated probe-sets private to AP offspring	No. of commonly regulated probe-sets of LP and AP offspring (intersection)
*94 dpc*					
12,650	7,937	1,001 (541 up, 460 down)			
*1 dpn*					
12,005	9,099	1 (0 up, 1 down)			
*28 dpn*					
12,307	8,250	483 (214 up, 269 down)			
*188 dpn*					
11,784	8,943	2,084 (952 up, 1,132 down)			
*developmental period 1*					
13,357	10,293	8,166 (3,731 up, 4,435 down)	1,042 (384 up, 658 down)	1,034 (503 up, 531 down)	6,090 (2,844 up, 3,246 down)
*developmental period 2*					
13,359	10,317	8,329 (4,118 up, 4,211 down)	881 (423 up, 458 down)	991 (448 up, 543 down)	6,457 (3,247 up, 3,210 down)
*developmental period 3*					
12,637	8,892	6,612 (2,810 up, 3,803 down)	1,959 (834 up, 1,125 down)	1,549 (697 up, 852 down)	3,104 (1,279 up, 1,825 down)

Developmental period 1 indicates the comparisons between stages 94 dpc and 1 dpn; Developmental period 2 indicates the comparisons between stages 1 dpc and 28 dpn; Developmental period 3 indicates the comparisons between stages 28 dpc and 188 dpn; 'Up' indicates higher expression values of later stages, 'down' indicates lower expression values at later stages.

### Comparisons between LP and AP within stages

**Figure 1:**
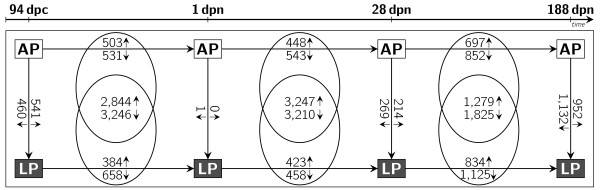
**Number of regulated probe-sets in liver tissue**. The numbers at the horizontal arrows indicate the quantity of probe-sets significantly regulated between the adjacent ontogenetic stages in either AP or LP offspring, whereas the numbers in the intersections indicate the quantity of probe-sets commonly regulated between stages in AP and LP offspring. The numbers at vertical arrows are the number of probe-sets differentially expressed between AP and LP offspring at the same ontogenetic stage. (Arrows between boxes show direction of the comparison; small arrows indicate up and down regulation, respectively).

Expression of mRNA was compared in LP and AP offspring within each ontogenetic stage (Figure 1). At stage 94 dpc 1,001 probe-sets differed significantly between LP and AP fetuses (541 LP ≻ AP). Ingenuity Pathway Analysis indicates enrichment of molecular routes related to genetic information and nucleic acid processing and cell cycle that were found to be diminished, whereas the 'Wnt signaling' was found to be increased in LP offspring (Table 2). In perinatal piglets (stage 1 dpn) 1 probe-set differed between LP offspring and AP offspring (0 increased). Therefore, no significant regulated metabolic pathway was determined.

**Table 2:** Significantly regulated transcripts of metabolic pathways in liver tissue within different ontogenetic stages (Ingenuity Pathway Analysis).

Ontogenetic stage	Regulated pathway	Direction of regulation	*P *value	No. of regulated genes	Genes involved in pathway
94 dpc	Wnt signaling	up	1.20*E-2	9	ACVR1, CSNK1G3, FZD4, FZD6, MAP3K7, MMP7, TCF4, TCF7L2, WNT5A
	Mitotic roles of Polo-like kinase	down	5.15*E-8	11	CCNB1, CCNB2, CDC23, CDC25B, CDK1, FZR1, SP90AA1, KIF11, PLK1, PPP2R1B, PTTG1
	G1/S checkpoint regulation	down	2.76*E-5	8	CCND2, CCND3, CCNE1, CDK4, E2F1, E2F4, RB1, TFDP1
	G2/M DNA damage checkpoint regulation	down	1.53*E-3	5	CCNB1, CCNB2, CDC25B, CDK1, PLK1
1 dpn	-	-	-	-	-
28 dpn	Complement system	up	2.07*E-5	5	C4B, C5, C6, C9, CD55
	G1/S checkpoint regulation	down	2.49*E-2	3	CDKN1B, HDAC11, TGFB3
188 dpn	VEGF signaling	up	2.80*E-4	12	ACTA2, AKT3, BCL2, EIF2B1, KDR, MAPK1, MRAS, PIK3R3, PRKCB, RRAS2, VCL, VEGFC
	mTOR signaling	up	6.29*E-3	13	AKT3, EIF3B, EIF4B, MAPK1, MRAS, PIK3R3, PPP2CB, PRKAB2, PRKCB, RHOJ, RRAS2, TSC1, VEGFC
	Synthesis and degradation of ketone bodies	up	1.18*E-3	4	ACAA1, BDH1, HADHA, HADHB
	Bile acid synthesis	up	3.33*E-3	7	ACAA1, ADH5, ADHFE1, ALDH7A1, HADHA, HADHB, LIPA
	Fatty acid elongation in mitochondria	up	4.81*E-3	4	ACAA1, AUH, HADHA, HADHB
	Glucocorticoid receptor signaling	up	3.99*E-2	17	AKT3, BCL2, CCL2, GTF2A2, GTF2B, GTF2E2, HSP90AB1, HSPA1B, IL1RN, MAPK1, MRAS, NCOR1, PBX1, PIK3R3, RRAS2, TAF4, VCAM1
	Val, Leu, Ile degradation	up	8.32*E-3	8	ACAA1, ACAD10, ALDH7A1, AUH, BCKDHB, HADHA, HADHB, HIBADH
	Biosynthesis of steroids	down	7.65*E-3	5	CYP24A1, CYP7B1, DHCR7, FDFT1, MVD

The comparison between the dietary gestational protein diets (LP vs. AP) is shown in dependence of the regulatory direction (up or down).

In juvenile piglets (stage 28 dpn) 483 probe-sets differed between LP and AP offspring. The expression of 214 probe-sets was increased in the LP offspring compared with AP offspring. Genes associated with the 'complement system' showed increased mRNA expression levels, whereas the canonical pathway 'G1/S checkpoint regulation' was found to be decreased in LP offspring.

At adult age (stage 188 dpn) 2,084 probe-sets were significantly different between LP and AP offspring. Of these, 952 probe-sets showed higher expression and 1,132 probe-sets showed lower expression in LP than in AP offspring. The mRNA expression levels of genes associated with metabolic processing of ketones, fatty acids, bile acids, and hydrophobic amino acids (Val, Ile, Leu) as well as 'mTOR signaling', 'VEGF signaling', and 'glucocorticoid receptor signaling', were increased in LP offspring at stage 188 dpn, whereas 'biosynthesis of steroids' was found to be diminished.

No genes were found consistently differentially expressed between the groups along all examined stages. However, at 94 dpc and 188 dpn 179 probe-sets were differentially regulated in both stages between LP and AP.

### Differences of longitudinal ontogenetic regulation among LP and AP offspring

Considering two adjacent ontogenetic stages within one treatment group, significantly regulated transcripts were determined. The resulting gene lists were compared between LP and AP offspring at the corresponding ontogenetic periods. The intersection of commonly regulated genes between those comparisons was discarded because regulation of these genes was likely due to physiologically developmental processes. Consequently, only genes whose regulation between two consecutive ontogenetic stages (period I: 94dpc-1dpn; period II: 1dpn-28dpn; period III: 28dpn-188dpn) was private to either the LP or the AP group were analysed (Table 1). These genes display diet-dependent longitudinal transcriptomic regulation (Figure 1). Thus, genes and pathways identified as regulated in one offspring group were either unregulated or showed an opposite direction of regulation in the corresponding ontogenetic period within the other dietary group.

Between fetal and perinatal stages (period I), there were 1,034 (503 1 dpn ≻ 94 dpc) probe-sets showing levels and directions of regulation in the AP group that were different from the LP group. Genes associated with 'AMPK signaling' were found to be increased at stage 1 dpn, while expression of genes associated with 'G2/M DNA damage checkpoint regulation', 'mitotic roles of Polo-like kinase' and 'pyrimidine metabolism' were decreased (Table 3). In LP offspring 1,042 probe-sets showed ontogenetic regulation (384 1 dpn ≻ 94 dpc) during the corresponding time period that was group-specific. The mRNA expression level of genes associated with cell cycle, mitosis, and metabolism of purines and pyrimidines was increased in LP offspring. Furthermore, genes participating in 'Wnt signaling' were found to be decreased in LP perinatal piglets.

**Table 3:** Significantly regulated transcripts of metabolic pathways in liver tissue between two ontogenetic stages within one dietary group (Ingenuity Pathway Analysis)

Ontogenetic comparison	Diet	Regulated pathway	Direction of regulation	*P *value	No. of regulated genes	Genes involved in pathway
94 dpc vs. 1 dpn	**AP**	AMPK signaling	up	6.96*E-3	8	AKT2, INSR, PIK3C2A, PIK3R1, PPM1B, PRKAA1, PRKACB, RAC1
	**AP**	Mitotic roles of Polo- like kinase	down	3.25*E-4	7	CCNB1, CCNB2, CDC25B, CHEK2, KIF11, PLK4, PTTG1
	**AP**	Pyrimidine metabolism	down	2.52*E-3	10	CAD, DCTD, DKC1, POLQ, POLR3E, POLR3K, RFC5, RRM1, TYMS, UCK2
	**AP**	G2/M DNA damage checkpoint regulation	down	2.04*E-4	6	CCNB1, CCNB2, CDC25B, CHEK2, UBC, YWHAE
	**LP**	Purine metabolism	up	9.20*E-3	12	DDX39, PNPT1, POLA2, POLE2, POLR1B, POLR1C, POLR2I, RFC3, RRM2, RRM2B, RUVBL1, RUVBL2
	**LP**	Pyrimidine metabolism	up	6.15*E-7	14	CTPS, PNPT1, POLA2, POLE2, POLR1B, POLR1C, POLR2I, PUS1, RFC3, RRM2, RRM2B, TXNRD1, TYMS, UCK2
	**LP**	Mitotic roles of Polo- like kinase	up	7.55*E-5	7	ANAPC4, CDC27, CDK1, ESPL1, FZR1, PPP2R1B, PTTG1
	**LP**	G2/M DNA damage checkpoint regulation	up	3.09*E-2	3	CDK1, CHEK1, YWHAZ
	**LP**	Wnt signaling	down	4.02*E-2	9	AKT3, FZD5, MMP7, NLK, SOX4, TCF3, TCF4, TCF7L2, WNT5A
1 dpn vs. 28 dpn	**AP**	AMPK signaling	up	4.26*E-3	8	AK1, CPT1A, EIF4EBP1, HMGCR, MAPK14, NOS3, PRKAA2, PRKAB2
	**AP**	mTOR signaling	up	1.89*E-2	7	EIF3F, EIF3G, EIF4EBP1, FNBP1, GNB1L, PRKAA2, PRKAB2
	**AP**	Val, Leu, Ile degradation	down	6.40*E-3	6	ACAD8, ACADL, ACADSB, BCAT1, DBT, MCCC2
	**LP**	Val, Leu, Ile degradation	up	7.12*E-8	11	ACADSB, ACAT1, ACAT2, ALDH1A1, AUH, BCKDHB, GCDH, HMGCL, HMGCS1, MCCC2, MCEE
	**LP**	Fatty acid metabolism	up	3.23*E-3	8	ACADSB, ACAT1, ACAT2, ALDH1A1, AUH, CYP51A1, GCDH, PECI
	**LP**	Synthesis and degradation of ketone bodies	up	1.76*E-6	5	ACAT1, ACAT2, BDH2, HMGCL, HMGCS1
	**LP**	Biosynthesis of steroids	up	3.27*E-4	5	CYP24A1, FDPS, HMGCR, IDI1, SC5DL
	**LP**	Glucocorticoid receptor signaling	up	3.57*E-3	12	CDKN1C, CXCL3, IL10, MAP3K1, NCOA2, NFKBIB, NR3C1, POLR2B, PRKACB, RRAS2, SLPI, SMARCA4
	**LP**	G1/S checkpoint regulation	down	2.22*E-2	4	CCNE1, CDC25A, CDKN1A, E2F3
28 dpn vs. 188 dpn	**AP**	AMPK signaling	up	1.50*E-3	10	INSR, MAPK12, PPAT, PPM1A, PPP2CA, PPP2R3A, PRKAA2, SMARCA2, SRC, STK11
	**AP**	Fatty acid metabolism	up	1.28*E-2	8	ACADSB, ALDH1A1, CYP1B1, CYP2D6, CYP3A4, CYP4A11, CYP4B1, PECI
	**AP**	Mitotic roles of Polo- like kinase	down	8.33*E-3	7	ANAPC5, CDK1, PLK1, PLK2, PPP2R1B, SLK, WEE1
	**AP**	VEGF signaling	down	7.3*E-3	9	AKT3, BCL2, KDR, NOS3, PRKCB, RAC2, RRAS, VCL, VEGFC
	**LP**	Val, Leu, Ile degradation	up	1.07*E-2	7	ACAA1, ACAT1, ALDH1B1, AUH, ECH1, IVD, IWS1
	**LP**	Fatty acid elongation in mitochondria	up	2.05*E-2	3	ACAA1, AUH, ECH1
	**LP**	mTOR signaling	up	2.65*E-2	10	AKT3, DDIT4, EIF4B, EIF4G3, PPP2R1B, PRKAB2, PRKAG1, PRKAG2, TSC1, VEGFA
	**LP**	Actin cytoskeleton signaling	down	7.99*E-5	24	ACTB, ACTR2, ARPC4, ARPC1A, CD14, CFL1, F2R, FGD1, LBP, MAP2K1,
						MYH9, NCKAP1, PAK2, PIK3C3, PIK3C2A, PIK3R4, PIKFYVE, PPP1R12A, RDX, ROCK1, ROCK2, TMSB4X, TMSL3, WASF1
	**LP**	RhoA signaling	dow	5.05*E-3n	12	ACTB, ACTR2, ARHGAP1, ARPC4, ARPC1A, CFL1, PIKFYVE, PPP1R12A, RDX, ROCK1, ROCK2, WASF1
	**LP**	Rac signaling	down	4.34*E-3	12	ACTR2, ARPC4, ARPC1A, CFL1, MAP2K1, NCKAP1, PAK2, PIK3C3, PIK3C2A, PIK3R4, PIKFYVE, WASF1
	**LP**	Complement system	down	2.17*E-2	5	C2, C7, C9, CFB, MBL2

The comparison between the dietary gestational protein diets (LP vs. AP) is shown in dependence of the regulatory direction (up or down).

Comparing perinatal and juvenile piglets (period II) 991 probe-sets were regulated in a different manner in AP offspring and in LP offspring. Of these, 448 probe-sets were up-regulated and 543 probe-sets showed lower expression at a higher age. Expression values of genes participating in 'AMPK signaling' and 'mTOR signaling' were increased, whereas genes associated with the 'degradation of valine, leucine and isoleucine' were decreased in AP offspring. In the same period 881 probe-sets exhibited ontogenetic regulation that was specific to LP offspring. Of these, 423 probe-sets showed an increased mRNA expression. Genes involved in 'fatty acid metabolism', 'biosynthesis of steroids', 'synthesis and degradation of ketone bodies', 'glucocorticoid receptor signaling' as well as the 'degradation of valine, leucine and isoleucine' were found to be up-regulated in LP offspring, while genes associated with 'G1/S checkpoint regulation' were down-regulated.

**Figure 2:**
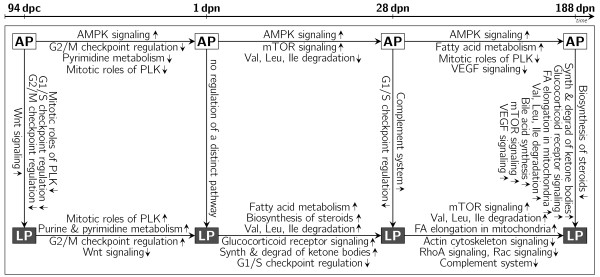
**Regulated pathways in liver tissue between ontogenetic stages and diets**. Listed pathways between AP stages (white boxes) indicate the appropriate ontogenetic development, which does not take place in the LP offspring (black boxes) at the corresponding developmental period. Pathways between the LP stages indicate processes and metabolic regulation, which occur in the LP offspring but not in the AP offspring in the corresponding developmental period. The differences in gene regulation dependent on diet and ontogenetic stage indicate fetal programming in terms of developmental and metabolic disorders (arrows between boxes show direction of the comparison; small arrows indicate up and down regulation, respectively; PLK, Polo-like kinase; VEGF, Vascular endothelial growth factor, mTOR, mammalian target of rapamycin; AMPK, AMP-activated protein kinase; RhoA, Ras homolog gene family, member A; Rac, Ras-related C3 botulinum toxin substrate; FA, Fatty acid)

When juvenile and young adult pigs (period III) are compared, 1,549 probe-sets differed significantly (697 188 dpn ≻ 28 dpn) in AP offspring. Genes participating in 'AMPK signaling' and 'fatty acid metabolism' were found to be up-regulated while genes associated with 'mitotic roles of Polo-like kinase' and 'VEGF signaling' were found to be down-regulated in AP offspring. In LP offspring 1,959 probe-sets were differently expressed (834 188 dpn ≻ 28 dpn). In LP offspring, genes involved in 'mTOR signaling', 'fatty acid elongation in mitochondria' as well as the 'degradation of valine, leucine and isoleucine' were up regulated. Furthermore, genes involved in 'actin cytoskeleton signaling', 'RhoA signaling', 'Rac signaling' and 'complement system' were found to be down-regulated. Figure 2 gives a comprehensive overview of the pathways found regulated between stages and diets. For all genes exemplarily analysed, qRT-PCR confirmed the direction of differential regulation as obtained by microarray analysis. In 90% of the genes that we validated by qRT-PCR, significant expression differences in mRNA levels between the treatment groups were identified by both qRT-PCR and microarray analysis (Table 4). Correlations between expression values of microarray and qRT-PCR of positive validated transcripts ranged between 0.47 and 0.86 and were highly significant. This suggests that our microarray data are reliable.

**Table 4:** Comparison of microarray data and qRT-PCR of selected transcripts

Gene name	Microarray			qRT-PCR #			Correlation ##
	**p-value**	**FC**	**Regulation**	**p-value**	**FC**	**Regulation**	**Expr. values**
*94 dpc*							
CCND2	0.022	-1.25	down	0.0001	-1.83	down	0.47 *
NCAPG	0.019	-1.73	down	0.003	-1.58	down	0.47 *
MGMT	0.031	+1.12	up	0.011	+1.32	up	0.53 **
GADD45B	0.669	-1.08	n.r.	0.522	+1.17	n.r	0.68 ***
*1 dpn*							
SDHB	0.0006	-1.19	down	0.028	-1.30	down	0.71 ***
CCND2	0.711	-1.06	n.r.	0.651	-1.09	n.r.	0.46 *
PPARGC1A	0.745	-1.03	n.r.	0.761	-1.04	n.r.	0.67 ***
*188 dpn*							
PRKAA1	0.0004	-1.68	down	0.027	-1.40	down	0.77 ***
PRKAA2	0.009	-1.36	down	0.095	-1.25	down	0.10
PPARGC1A	0.126	-1.35	n.r.	0.105	-1.36	n.r.	0.86 ***

*CCND2 *- cyclin D2; *NCAPG *- non-SMC condensin I complex, subunit G; *MGMT *- O-6-methylguanine-DNA methyltransferase; *GADD45B *- growth arrest and DNA-damage-inducible, *β *; *SDHB *- succinate dehydrogenase complex, subunit B, iron sulfur (Ip); *PPARGC1A *- Peroxisome proliferator activated receptor *γ *coactivator-1*α*; *PRKAA1 *- 5'-AMP-activated protein kinase, catalytic *α*1 chain; *PRKAA2 *- 5'-AMP-activated protein kinase, catalytic *α*2 chain *calculated by factorial normalisation on *RPL10 *expression values; **p-value of pamn' rho; n.r. - not regulated

## Discussion

We applied whole-genome microarrays to evaluate hepatic gene expression profiles of offspring from sows fed either an isocaloric maternal low protein or adequate protein diet throughout their pregnancy. In order to investigate transcriptional features of developmental nutritional programming we conducted a longitudinal experimental design covering prenatal, perinatal, juvenile and adult ontogenetic stages in a porcine model. The comparisons of the relative mRNA abundances depending on dietary group and ontogenetic stages provide an overall view of the developmental plasticity of the liver.

At the prenatal stage mRNA expression profiles were extensively altered between the dietary groups. At this time point the fetuses were subjected to a serious nutritional deficiency that requires an acute transcriptomic response. Following in utero exposure to a gestational low protein diet at 1 dpn we could not find significant regulatory changes of genes of any molecular route in porcine perinatal liver. At this stage the animals experienced a kind of release from metabolic burden after cross-fostering and suckling. Obviously, this initiates an immediate recovery of the activity of genes to 'normal level' reflected by similar expression pattern in LP and AP piglets. However, at the whole-body level adverse consequences of restricted intrauterine supply were observed [15]. In juvenile LP offspring, mRNA expression profiles were moderately altered compared to AP offspring. However, the number of differentially expressed probe-sets increased extensively at adult stage. The observed transcriptional postnatal regulations were delayed long-term effects of the prenatal nutritional supply. Due to the normal dietary conditions at juvenile and adult stages the alterations found here can be regarded as fetal nutritional programming.

Our model indicates that gestational LP diets affected the hepatic expression profiles in an acute, short-term as well as in a delayed, long-term manner in LP offspring. Due to the LP diet a number of molecular routes related to cell cycle and cellular turnover, response to stimuli, as well as energy-, lipid- and amino acid metabolic pathways are shifted on the transcriptional level in the liver at prenatal stages under the direct influence of limited protein and/or amino acid supply, but also postnatal. The effect is characterised by a programming of the genome that leads to different responsiveness and adaptability of the gene expression machinery to chronic and acute environmental stimuli, i.e. nutritional supply. Together with the observation that the offspring of both groups did not differ in bodyweight but in body composition and fat content [17], the data indicate that the activity of different genes led to different partitioning of nutrients among peripheral and visceral organs and tissues.

### Transcriptional excursions regarding cell maintenance and proliferation

Mammalian cell division is precisely regulated by variate factors and functional networks. Therefore, cell division is synchronous with cell growth [19,20]. Eukaryotic cells evolved elaborate mechanisms to verify the fidelity of cell division. Therefore, a cell cycle control system can arrest the cycle at certain checkpoints. Key components in terms of cell cycle regulation are cyclins. Cyclin levels undergo an oscillation of synthesis and degradation in each cell cycle and fall at a proper developmental time point to exit the cell cycle [21,22]. Therefore, the transcriptional control of cyclins provides an additional level of growth regulation. Mitotic cyclins interact with Polo-like kinases (PLK), an evolutionary conserved family of essential cell cycle regulators, which are required at several key points within mitosis, including entry into and exit from mitosis [23,24]. It has been shown that fetal growth retardation is accompanied by alterations in cell cycle regulating molecules at both transcriptome [25] and proteome level [26]. However, longitudinal studies focussed on diet-dependent alterations of cell cycle regulators are scarce. The porcine expression patterns in this study showed a decreased mRNA expression of genes associated with the control of mitosis and cell cycle checkpoint regulation at stage 94 dpc in LP fetuses. These regulations may indicate an impaired fetal growth performance due to the gestational low protein diet. Therefore, the down-regulation of cell cycle regulating pathways might lead to a lengthened cell cycle in LP fetuses and offspring [27] and cumulate in growth retardation in LP fetuses. In AP fetuses, cell cycle parameters were found to be decreased in expression within developmental period I, which indicates a terminated fetal growth. In contrast, LP fetuses showed an increased expression of transcripts associated with cell division within the corresponding developmental period. Obviously, the increased mRNA expression of genes related to cell cycle regulators within developmental period I in LP offspring accounts for compensatory regulations regarding the lowered fetal weight due to the gestational diet. Within developmental period III, AP offspring showed a decreased expression of genes associated with Polo-like kinases, which is in line with the assumption that LP pigs have not terminated their growth at stage 188 dpn; in particular their liver has not attained a steady-state and that there is persistent functional demand towards cell proliferation potentially to respond to an elevated metabolic burden, including a higher turn over of lipids and ketone bodies.

The expression level of *NCAPG *(non-structural maintenance of chromosomes (SMC) condensin I complex subunit G) was highlighted as strongly associated with fetal growth retardation [28,29]. According to recent studies *NCAPG *is important during mitotic cell division. Due to the gestational low protein diet *NCAPG *was found to be down-regulated in LP fetuses compared to AP fetuses, which reflects the organismal effort to counteract the growth retarding processes in LP fetuses.

Beside pathways related to the control of growth and cell deviation, the diet-dependent expression patterns showed regulation in genes associated with biosynthesis, degradation and salvage of nucleotides ('purine and pyrimidine metabolism') as well as cell motility and cytokinesis ('actin cytoskeleton signaling', 'Rac signaling', 'RhoA signaling'). These expression patterns suggest a dietary effect on cellular turn-over, which might be increased at early stages but decreased at adult stage in LP offspring. Therefore, a diet-dependent tissue remodelling might take place in LP offspring as observed previously [30].

The 'mTOR signaling' acts as an important nutrient sensing pathway that controls protein synthesis in mammalian cells at the level of translation [31]. Upstream signaling events of 'mTOR signaling' include alterations in amino acid availability, abundance of hormones, AMP and growth factors [32]. Thus, 'mTOR signaling' is involved in regulating individual cell growth, growth performance, and developmental processes [33,34]. An increase of 'mTOR signaling' within developmental period II was observed in AP offspring, but not in the LP group. This may account for improper protein synthesis [35] in LP offspring. However, at adult stage a diet-dependent up-regulation of 'mTOR signaling' in LP offspring suggests a transcriptional priority for cellular growth and proliferation. Therefore, 'mTOR signaling' might be involved in compensatory growth processes at adult stage in LP offspring.

Another signaling pathway identified to be regulated in a diet-dependent manner is the 'VEGF (vascular endothelial growth factor) signaling' pathway, which is known from gene deletion studies to be essential in developmental processes [36,37]. Therefore, the transcriptional up-regulation of 'VEGF signaling' reflects the effort on cell maintenance and angiogenic growth in LP offspring at stage 188 dpn. Obviously, hepatic ontogenetic growth is not finished at adult stage in LP offspring in contrast to AP offspring.

Furthermore, the dietary modifications led to regulation of the 'Wnt signaling' pathway, which is involved in various aspects of embryogenesis, including cell differentiation and cell proliferation [38]. Therefore, the observed mRNA expression patterns might contribute to an impaired developmental growth in LP offspring.

Regarding the LP model used in rodents, it has been suggested that the maternal LP diet had an impact on 'insulin signaling' as well as on 'IGF1 signaling' and its binding proteins [30,39-43]. However, in this study no regulation of both 'insulin signaling' and 'IGF1 signaling' was observed in liver tissue of LP porcine offspring, possibly due to maturation-, species-, tissue-, sex- and time-dependent regulations.

### Transcriptional excursions regarding lipid-, energy- and N-metabolism

During postnatal development a remarkable number of diet- and stage-dependent transcriptionally regulated pathways were related to lipid metabolism, including 'fatty acid metabolism', 'fatty acid elongation in mitochondria', 'synthesis and degradation of ketone bodies', 'bile acid synthesis' and 'biosynthesis of steroids'. These findings suggest that pathways associated to lipid metabolism are part of postnatal adaptive responses to the prenatal nutritional environment in LP offspring. Consistent with these observations, genes associated with lipid metabolism were found to be altered at pre- and postnatal stages in rodents, where the LP model was studied intensively [44-49]. Furthermore, the transcriptional adaptations may have consequences for the offspring's phenotype, including alterations of plasma parameters [44-46,50], hepatic histology [46] and body composition [47].

According to the longitudinal study design the 'AMPK (AMP-dependent activated kinase) signaling' was found to be regulated throughout ontogenesis in AP offspring only. 'AMPK signaling' is a metabolic pathway which is involved in the regulation of the lipid and energy metabolism in mammalian cells [51-56]. In particular, the cellular energy sensor AMPK is essential for a proper mitochondrial activity and metabolic health citeTowler2007. Therefore, the up-regulation of 'AMPK signaling' in developmental periods I, II and III in AP offspring accounts for metabolic health in AP offspring, but not in LP offspring. On the one hand, this matter may be related to mitochondrial impairments which may happen due to the LP diet, as has been suggested previously [57,58]. On the other hand, it might be associated to the transcriptional excursions within the lipid metabolism in LP offspring. Moreover, the expression of the catalytic subunits of AMPK, *PRKAA1 *and *PRKAA2 *was found to be decreased in LP offspring compared to AP offspring at stage 188 dpn, which may contribute to an imbalanced cellular metabolism. Taken together, the observed regulations were seen as a clue for impairments in energy metabolism in LP offspring during ontogenesis.

Furthermore, the diet-dependent transcriptional excursions revealed an impact on N-metabolism. In particular, the degradation of essential, branch-chained amino acids like valine, leucine and isoleucine was found to be regulated, which may illustrate the LP offspring's efforts to mimic prenatal experiences in terms of dietary supply during postnatal development in the face of richer conditions. Although essential amino acids are needed for growth, anabolic and developmental processes, transcriptional levels of genes leading to degradation of those dietary valuable compounds is forced during postnatal development in LP offspring.

### Transcriptional excursions regarding stress and immune response

The biological effect of glucocorticoids is modulated by glucocorticoid receptors. By means of succeeding direct and indirect interactions of downstream target genes, mRNA expression regarding metabolic, behavioural, cardiovascular and immune functions is modulated. In rodents, many studies reported an impact of maternal low protein diets on expression and affinity of the glucocorticoid receptor and its signaling molecules [8,48,59-61]. Consistently, in porcine offspring a diet-dependent regulation of 'glucocorticoid receptor signaling' was also found. Therefore, expression profiles at weaning and adult stages point to an alarm alert (activated defence) in LP offspring. Furthermore, genes associated with the complement system were found to be regulated in a diet-dependent manner at stage 28 dpn. Obviously, at least a part of the innate immune system is impaired when LP offspring is faced with a pre-biotical stress like weaning. Consistent with this finding, expression of genes associated with an impaired immunity were found to be altered in offspring of protein malnourished dams [62,63].

## Conclusions

In conclusion, the longitudinal survey of the hepatic transcriptomic response in offspring of dams fed either a gestational low protein diet (LP, 6% CP) or an adequate protein diet (AP, 12% CP) at prenatal (94 dpc), perinatal (1 dpn), juvenile(28 dpn), and adult (188 dpn) ontogenetic stages revealed acute short-term and delayed long-term modulations. The changes in gene expression were not persistent in terms of consistent differential expression of genes at all stages. However, genes related to cell cycle and cellular turnover were differentially expressed at all stages except stage 1 dpn. This stage appeared as a phase of recovery of the activity of genes. Differential expression of genes related to lipid, ketone body, and amino acid metabolism indicate that the offspring of both groups uses different metabolic directions in response to identical nutritional condition during postnatal life.

## Methods

### Animals and sample collection

**Figure 3:**
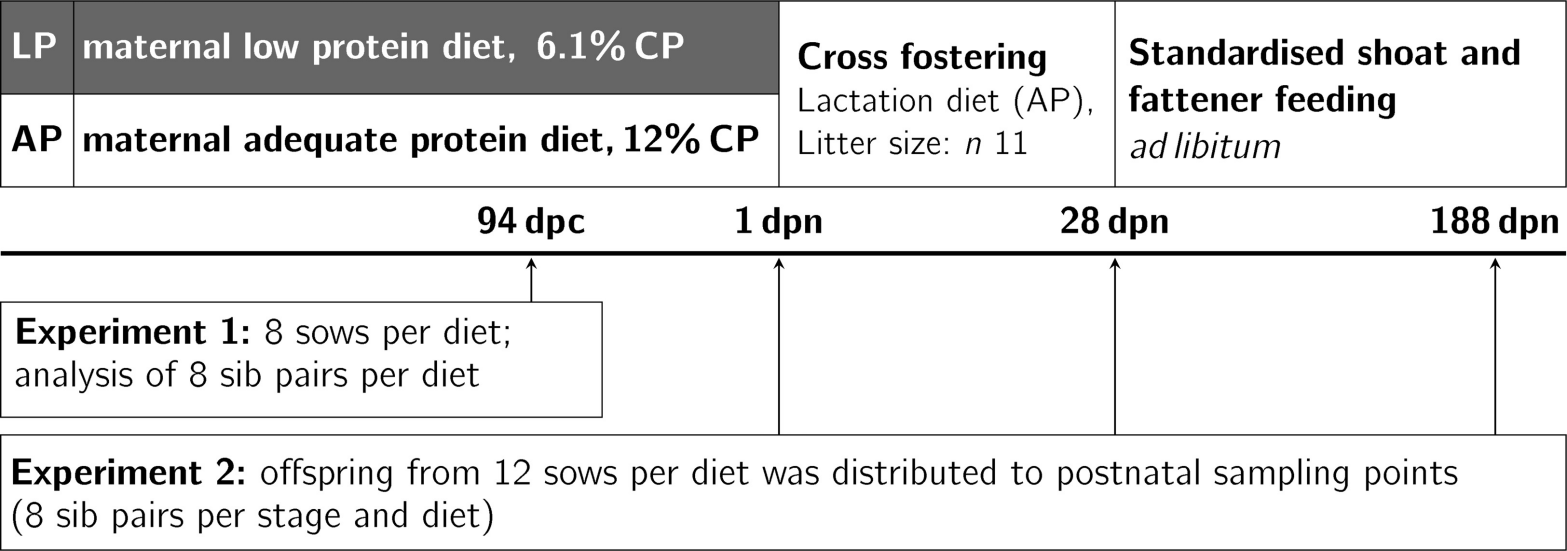
**Experimental design**.

Animal care and tissue collection processes followed the guidelines of the German Law of Animal Protection and the experimental protocol was approved by the Animal Care Committee of the State Mecklenburg-Vorpommern (Landesamt für Landwirtschaft, Lebensmittelsicherheit und Fischerei, Mecklenburg-Vorpommern, Germany; LVL MV/TSD/7221.3-1.1-006/04; LALLF M-V/TSD/7221.3-1.2-05/06; LALLF M-V/TSD/7221.3-1.2-013/06). The animal experiment was performed as described [15]. Briefly, at insemination German Landrace primiparous sows (n = 42) were randomly assigned to either a low protein diet (LP) with 6% (w/w) crude protein or an adequate protein diet (AP) containing 12% crude protein. Diets were formulated to be isocaloric (13.6 MJ ME/kg on average) [15]. Tissue sampling included offspring of these sows at one prenatal (94 dpc) and three postnatal (1, 28, 188 dpn) time points (Figure 3).

At 94 dpc of gestation, a subset of eight sows per dietary group was subjected to Caesarean section (EXP1). Sows were anaesthetised as described [15]. This experiment was performed over 5 replicates. Eight viable fetuses per sow were collected starting at the tip of the left uterus horn and alternating between left and right horn. Fetuses were killed by i.v. injection of T61 in the V. cava cranialis and liver samples were immediately collected (approximately 500 mg), frozen in liquid nitrogen, and stored at -80°C until analysis. Fetuses originated from litters of at least 11 viable piglets. Fetuses of LP-fed dams showed a numerically lowered weight compared to AP fetuses at 94 dpc (LP: 661 ± 115 g, and AP: 711 ± 118 g, respectively; *P *= 0.12; n = 32). The smallest and the heaviest fetus within one litter were selected for transcriptome analysis.

In the second experiment (EXP2) offspring selected for the postnatal time points was born to primiparous sows after prostaglandin induction of parturition as described [15] and farrowed after mean pregnancy duration of 115 days. This experiment was conducted over 8 replicates and offspring of a subset of 4 sows (2 per diet per replicate) with a minimum of 11 live born piglets (median litter size = 13) were used. At birth 10 piglets in each litter were distributed over three time points (1, 28, 188 dpn). For the microarray analyses, 8 sib pairs (light and heavy piglet from one litter) which were balanced for sex (all stages) and discordant for weight (light and heavy piglet; stages 94 dpc and 1 dpn only) were chosen per stage and diet.

Mean birth weights of LP newborn piglets used for microarray analyses were numerically lower than birth weights of AP offspring (LP: 1.21 ± 0.30 kg, AP: 1.36 ± 0.31 kg, *P *= 0.08, n = 32). Including all piglets derived from the complete experiment, LP offspring was growth-restricted compared to AP offspring, but litter size did not differ [15]. Thirty-six hours after birth, the lightest and the heaviest piglet within one litter were killed by i.m. injection of 1.25 mg propionyl-promazine (0.2 ml Combelen, Bayer AG, Leverkusen, Germany) and 50 mg ketamine (Ursotamin, Serumwerk Bernburg AG, Germany). Samples were immediately collected from lobus sinister hepaticus (approximately 500 mg), frozen in liquid nitrogen, and stored at -80°C until analysis.

The remaining piglets were cross-fostered to non-experimental sows of 2^nd ^to 4^th ^parities, which were on the AP diet during gestation. All sows were fed AP lactation diets. Litter size during suckling was standardised to 11 piglets per sow. Male piglets were castrated at d 4 of age. From weaning (28 dpn) to slaughter (188 dpn), all piglets were individually reared. They had free access to standard diets formulated for post-weaning (d 29 to d 76), growing (d 77 to d 105) and finishing periods [64]. At 28 dpn and 188 dpn of age, pigs were weighed after an overnight fast and killed by electronarcosis followed by exsanguination in the experimental slaughterhouse of FBN. Among the animals used for the microarray expression analysis, weaners of the LP group showed numerically but not significantly lower body weight than the AP group, (LP: 7.24 ± 1.56 kg, AP: 7.59 ± 2.14 kg, *P = *0.30, n = 32). At 188 dpn the body weight was slightly but significantly reduced in LP compared to AP (LP: 123.19 ± 9.15 kg, AP: 131.55 ± 15.11 kg, *P *- 0.03; n - 32). For all animals of the experiment no significant differences in body weight were observed at 28 and 188 dpn [15,16]. Liver tissue was immediately collected from lobus sinister hepaticus, frozen in liquid nitrogen, and stored at - 80°C until use for RNA isolation.

### RNA isolation, target preparation and hybridisation

According to the manufacturer's protocol total RNA from individual liver samples was isolated using Tri-Reagent (Sigma-Aldrich, Taufkirchen, Germany). After DNase treatment a column-based purification using the RNeasy Mini Kit (Qiagen, Hilden, Germany) was done. The RNA samples were visualised on 1% agarose gels containing ethidium bromide to check RNA integrity. RNA was quantified by spectrometry with a NanoDrop ND-1000 spectrophotometer (PEQLAB, Erlangen, Germany). To ensure the absence of a DNA contamination within the isolated RNA a PCR amplification with the porcine glyceraldehyde-3-phosphate dehydrogenase (*GAPDH*) gene was done (Forward primer: aagcagggatgatgttctgg; Reverse primer: atgcctcctgtaccaccaac). All RNA samples were stored at -80°C until downstream analysis was performed. For the microarray experiments individual biotin-labeled cRNA was synthesised by the Gene Chip 3' Express Kit (Affymetrix, Santa Clara, CA, USA). The cRNA was fragmented (≈ 100 bp) and hybridised on Affymetrix GeneChip^®^porcine Genome Array. After staining and washing steps the arrays were scanned (Affymetrix, Santa Clara, CA, USA).

### Data analysis

The bioinformatic analysis was done in R [65]. Firstly, a quality control was performed. At 94 dpc 15 AP-samples met the appropriate quality control criteria (94 dpc-LP: 16; 1 dpn-AP: 15; 1 dpn-LP: 14; 28 dpn-AP: 14; 28 dpn-LP: 15; 188 dpn-AP: 16; 188 dpn-LP: 16 samples). Samples were GC-RMA normalised (Log2). The MAS5 algorithm was used to skip those transcripts which were expressed in less than 50% of the animals within one dietary group per stage. For a second filtering step standard deviations were calculated for each probe-set over all subsets of arrays of the particular comparisons. Probe-sets with a low standard deviation (*s *≤ 0.25) were discarded, because such transcripts are not likely to be regulated.

Relative changes in mRNA levels were determined using a mixed model analysis, including effects of dietary treatment, sex, mother, birth weight and interaction between diet and sex (*v_ijkl _*= *diet_i _*+ *sex_j _*+ *mother_k _*(*diet_i_*) + *weight_l _*+ (*dietxsex*)_*ij *_+ *error_ijkl_*). *P*-values (significance set at *P ≤ *0.05) for each stage were converted to a set of q-values (*q *≤ 0.25) using the algorithm proposed by Storey and Tibshirani [66]. In general, results are given for the comparisons in the direction of LP vs. AP; thus 'up-regulation' or 'increased expression' indicates higher expression in LP than in AP. Analysis of the pathways involved was carried out using Ingenuity Pathway Analysis [67]. The up-to-date annotation of Affymetrix probe-sets to EnsEMBL Sscofa 9 (20,439 of 23,935 annotated probe-sets) was used [18]. All the microarray data is MIAME compliant and the raw data has been deposited in a MIAME compliant database, the National Center for Biotechnology Information Gene Expression Omnibus http://www.ncbi.nlm.nih.gov/geo (accession numbers: GSE25482 and GSE31191).

### Pathway analysis

Gene lists from microarray results were submitted to the manually curated database 'Ingenuity Pathways Analysis' to elucidate putative pathways associated with an altered gene expression in porcine liver. The focus was on those canonical pathways which came up at least once within the top ten regulated pathways within one single analysis. It should be noted here that the interactions presented in the networks are not specific for porcine liver tissue, as the database contains literature from many different research areas.

### Quantitative real-time RT-PCR

First-strand cDNA was synthesized from 2*μg *of total RNA (n = 14 per diet and stage) using random primers and oligo d(T) 13VN in the presence of Superscript III reverse transcriptase (Invitrogen, Karlsruhe, Germany). In order to survey expression of the liver tissue samples, total transcript levels of selected target and reference genes (Table 5) were quantified by real-time quantitative PCR (qPCR) performed on a LightCycler^®^480 system using the LightCycler 480 SYBR Green I Master(Roche, Mannheim, Germany). The amplification was conducted in duplicate according to manufacturer's instructions using 10 *μM *of each primer. Reactions were performed in a final volume of 10*μl *using 5.0*μl *of LightCycler 480 SYBR Green I Master (Roche), 2.0 *μl *of *Aqua dest*., 10 *μM *(0.5 *μl*) of each primer (Table 5) and 40 ng (2 *μl*) cDNA. The temperature profiles comprised an initial denaturation step at 95°C for 10 min and 40 cycles consisting of denaturation at 95°C for 15 s, annealing at 60°C for 10 s and extension/fluorescence acquisition at 72°C for 15 s. For all the assays threshold cycles were converted to copy numbers using a standard curve generated by amplifying serial dilutions of an external PCR standard (10^7 ^- 10^2 ^copies). At the completion of the amplification protocol, all samples were subjected to melting curve analyses and gel electrophoresis to verify the absence of any non-specific product. To account for variation in RNA input and efficiency of reverse transcription the calculated mRNA copy numbers were normalized by dividing with a normalization factor derived from the expression of the reference gene. In total, 28 individual liver mRNA samples were analyzed in duplicate per stage. Data were analyzed using the PROC MIXED, including effects of diet, sex, mother, birth weight and interaction between diet and sex (SAS version 9.1; SAS Institute, Cary, NC). Differences were considered significant at *P *≤ 0.05.

**Table 5:** Primer used to verify microarray experiments in liver tissue by qRT-PCR

Gene name	Probe-set ID	Sequence 5' - 3'	T(°C)	Size (bp)
CCND2	Ssc.15749.1.S1_at	For AGGAGCAGATTGAGGTCGTG	86	185
		Rev CAACCAGAGAGAAGGAAGGAGA		
NCAPG	Ssc.28512.1.S1_at	For CTTGTAGATTTGACGAGACCA	60	156
		Rev GGCTTTAGTATAGACCCGAAC		
MGMT	Ssc.19639.1.A1_at	For GCAACTACTCGGGAGGAATG	88	171
		Rev CTGCGAACGCTCAGTCTTG		
GADD45B	Ssc.14764.1.A1_at	For GGACTTAGACTTTGGGACTTG	60	140
		Rev GTAAGCCTCCCATCTCTCTT		
SDHB	Ssc.8939.1.S1_at	For GCAGGACCCGTTCTCTCTGT	60	170
		Rev GGTTACAGTCACGTTAGGTTGG		
PPARGC1A	Ssc.16864.1.S1_at	For GTAAATCTGCGGGATGATGG	60	208
		Rev TGGTGGAAGCAGGATCAAAG		
PRKAA1	Ssc.8107.1.A1_at	For TTGTTAATTTCATAAACTTTGCTTC	60	193
		Rev GTGCAGCCTTGACATACTC		
PRKAA2	Ssc.16257.1.S1_at	For TCTGTAATTCTGTTTTGCCTACGA	60	168
		Rev AGCAAGAAGGTGATGCCAAG		
RPL10*	Ssc.9130.1.A1_at	For CTGTGTTCGTCTTTTCTTCC	60	199
		Rev TCATCCACTTTTGCCTTCT		

*CCND2 *- cyclin D2; *NCAPG *- non-SMC condensin I complex, subunit G; *MGMT *- O-6-methylguanine-DNA methyltransferase; *GADD45B *- growth arrest and DNA-damage-inducible, *β *; *SDHB *- succinate dehydrogenase complex, subunit B, iron sulfur (Ip); *PPARGC1A *- Peroxisome proliferator activated receptor *γ *coactivator-1*α*; *PRKAA1 *- 5'-AMP-activated protein kinase, catalytic *α*1 chain; *PRKAA2 *- 5'-AMP-activated protein kinase, catalytic *α*2 chain; *RPL10 *- Ribosomal protein 10; * house keeping gene.

## Competing interests

The authors declare that they have no competing interests.

## Authors' contributions

Conceived and designed the experiments: KW CCM SP. Performed the experiments: MO EM CCM SP KW. Analyzed the data: MO EM SP KW. Contributed reagents/materials/analysis tools: CCM KW SP EM. Wrote the paper: MO KW. All authors read and approved the final manuscript.
